# Variability of inter-syllable gaps challenges the branched-chain model of sequence production in Bengalese finches

**DOI:** 10.1186/1471-2202-13-S1-P19

**Published:** 2012-07-16

**Authors:** Kristofer E Bouchard, Anand S Kulkarni, Michael S Brainard, Todd W Troyer

**Affiliations:** 1 Department of Physiology, UCSF, San Francisco, CA 94143, USA; 2 Biology Department and Neurosciences Institute, UTSA, San Antonio, Texas, 78249, USA; 3 Department of Neurosurgery, UCSF, San Francisco, CA, 94143, USA

## 

Songbirds have emerged as a premier model system for studying how brain circuits learn and produce complex action sequences. The adult song of the most widely studied songbird, the zebra finch (ZF), consists of repeats of a stereotyped sequence of vocal gestures known as syllables. These songs are incredibly precise, with individual syllables and inter-syllable gaps varying in length by roughly 5% (std. dev.). Electrophysiological recordings in singing birds reveal that song related neural activity is also precise, with individual neurons in the premotor nucleus HVC producing one burst of action potentials per song sequence, locked to song acoustics with sub-millisecond precision [1]. This precision and reliability has led to the suggestion that the HVC circuit is organized as a synfire chain, with activity propagating down a chain-like network of strongly connected groups of neurons [2].

**Figure 1:**
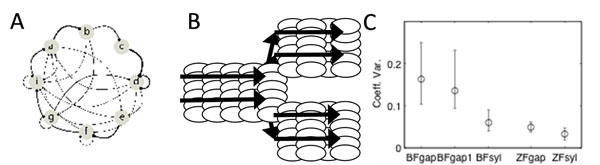
Variable sequencing of Bengalese finch song **A**. Transition diagram for single BF; strength of the arrow is proportional to transition probability. **B**. Branched synfire chain model. **C**. Median and inter-quartile range of coefficient of variation for BF gaps, BFgaps without branching (transition prob = 1), BF syls, and ZF gaps and syls.

Here we examine the songs of a closely related species, the Bengalese finch (BF). These birds also learn sequences of highly stereotyped syllables. However, sequencing is variable and includes ‘branching’ in which some song syllables can be followed by more than one (2-4) subsequent syllables (fig1A). Given the many similarities between species, it has been hypothesized that variable sequencing in BF is accomplished by a branched synfire network in HVC [3] (fig1B). Such a model suggests that timing precision in BFs should be similar to that of ZFs, perhaps with a bit of added variability due to the competition between branches. However, this added variability should not be longer than the maximum latency between successive links in the synfire chain (~20 msec).

To test this, we measured the durations of both syllables and inter-syllable gaps in a large sample of BF songs (32 birds, 52,451 transitions between 303 unique syllable pairs; syllables were hand labeled by visual inspection and durations were determined by a hand-set threshold optimized for each bird.) Overall, the mean and coefficient of variation (CV) for BF inter-syllable gap durations were qualitatively more variable than for BF syllables, ZF syllables or ZF gaps, even at syllable transitions that were not branched (fig 1C). These results contradict the simplest branched synfire chain model of variable sequencing in BFs, and provide significant challenges for more general models based on this idea.
